# ARBic: an all-round biclustering algorithm for analyzing gene expression data

**DOI:** 10.1093/nargab/lqad009

**Published:** 2023-01-31

**Authors:** Xiangyu Liu, Ting Yu, Xiaoyu Zhao, Chaoyi Long, Renmin Han, Zhengchang Su, Guojun Li

**Affiliations:** Research Center for Mathematics and Interdisciplinary Sciences, Shandong University, Jinan 250100, China; Research Center for Mathematics and Interdisciplinary Sciences, Shandong University, Jinan 250100, China; Research Center for Mathematics and Interdisciplinary Sciences, Shandong University, Jinan 250100, China; Research Center for Mathematics and Interdisciplinary Sciences, Shandong University, Jinan 250100, China; Research Center for Mathematics and Interdisciplinary Sciences, Shandong University, Jinan 250100, China; Department of Bioinformatics and Genomics, The University of North Carolina at Charlotte, Charlotte, NC 28223, USA; Research Center for Mathematics and Interdisciplinary Sciences, Shandong University, Jinan 250100, China; School of Mathematical Science, Liaocheng University, Liaocheng 252000, China

## Abstract

Identifying significant biclusters of genes with specific expression patterns is an effective approach to reveal functionally correlated genes in gene expression data. However, none of existing algorithms can simultaneously identify both broader and narrower biclusters due to their failure of balancing between effectiveness and efficiency. We introduced ARBic, an algorithm which is capable of accurately identifying any significant biclusters of any shape, including broader, narrower and square, in any large scale gene expression dataset. ARBic was designed by integrating column-based and row-based strategies into a single biclustering procedure. The column-based strategy borrowed from RecBic, a recently published biclustering tool, extracts narrower biclusters, while the row-based strategy that iteratively finds the longest path in a specific directed graph, extracts broader ones. Being tested and compared to other seven salient biclustering algorithms on simulated datasets, ARBic achieves at least an average of 29% higher recovery, relevance and$\ {F}_1$ scores than the best existing tool. In addition, ARBic substantially outperforms all tools on real datasets and is more robust to noises, bicluster shapes and dataset types.

## INTRODUCTION

With the development of high-throughput sequencing technology, a large amount of gene expression data have been produced using RNA-seq technologies (1). These massive expression data provide an opportunity to find co-expressed genes under specific conditions, which form bases for various functional analyses, including associating genes of unknown functions with biological processes, prioritizing candidate disease genes, and discerning transcriptional regulatory networks (2). Traditional gene clustering algorithms attempt to find groups of genes that are co-expressed across all conditions or tissues. However, if genes are co-expressed only under certain conditions or in certain tissues, biclusters that are formed by genes under certain conditions or in tissues should be identified as they are more appropriate to represent such co-expressed genes.

Mathematically, biclustering is to find a submatrix in a real number matrix where the rows of the submatrix are correlated under the columns of the submatrix. Identifying gene expression patterns under different conditions (such as cancerous tissues at different pathological stages), one may find genes involved in specific biochemical pathways responsible for cellular phenotypes, or dysregulated during the development of cancer. The biclustering problem can be traced back to Morgan *et al.* (3) and Hartigan (4) who attempted to partition a numerical matrix into submatrices whose values are as similar as possible. Since Cheng and Church (5) first introduced a biclustering algorithm for gene expression data analyses, numerous biclustering algorithms (6–13) have been developed. However, there are still many challenges in solving the general biclustering problem (14,15).

The first challenge is to define biclusters such that they better represent genes that are functionally related. Chen and Church (5) sought to find the biclusters with low variance, as defined by the mean squared residual. However, this definition is only suitable for finding constant, row constant, column constant and shift biclusters. As the function-related genes may have some scalable relationships in their expression, various other definitions of biclusters (Supplementary Figure S1) have been proposed, such as scale, shift + scale, and trend-preserving patterns (14,16). The biclusters of trend-preserving patterns are more inclusive and more biologically meaningful (14,16). A bicluster is said to be trend-preserved if and only if any pair of rows/genes within the bicluster are trend-preserved. Two rows are said to be trend-preserved if and only if they are either order-preserved or order-reversed. Two rows (vectors) *x* and *y* are said to be order-preserved if and only if any two corresponding components have the same rank (with respect to the numerical value) in their respective rows, and order-reversed if and only if *x* and -*y* are order-preserved (16). Although many biclustering algorithms have been developed to find order-preserving biclusters, none of them is able to handle both broader and narrower biclusters (17).

The second challenge is to define an objective function on biclusters whose optimization may lead to accurate identification of biclusters. Although many objective functions have been proposed, most of them suffer high false discovery rate to recognize co-expressed genes under certain conditions. For instance, CC (5) and UniBic (16) simply used bicluster size as their objective function; OPSM (18) and EBIC (14) defined an objective function based on the probability that a bicluster is randomly generated; QUBIC2 (19) employed the KL score as its objective function based on the difference between the distributions of elements in the bicluster to be tested and in the data matrix. Therefore, it is imperative to develop a new objective function for extracting all co-expressed genes under certain conditions with the false discovery rate as small as possible.

The third challenge is to design an efficient algorithm to optimize the objective function. As the biclustering problem has been proved to be NP-hard even for the binary data matrix (20), we thus have to focus on heuristic algorithms. There have been quite a few probabilistic algorithms, such as Plaid (21), FABIA (22), ISA (23), CC (5), BBC (8), which are computationally efficient to find broader biclusters, i.e. with many columns but only few rows, but they are all inefficient to find narrower ones, i.e. with many rows but only few columns. On the other hand, there have been many more heuristic algorithms, such as xMOTIFs (24), CPB (10), UniBic (16), QUBIC (25) and QUBIC2 (19), which perform well in accuracy, but are not computationally efficient and incapable of coping with narrower biclusters either. Very recently, two algorithms EBIC (14) and RecBic (26) were developed specifically to identify narrower biclusters, but both fail to find broader biclusters.

To overcome these limitations, we designed a new algorithm, termed ARBic, which is capable of extracting both narrower and broader biclusters in a large data matrix based on a new objective function and a graph theoretic optimization procedure, and compensates for the shortage inherited from UniBic (Supplementary Figure S2). ARBic identifies broader biclusters by iteratively finding the longest path in a specific directed graph, and it finds narrower ones by calling RecBic when the number of columns of the data matrix is <500, because we have shown that RecBic works well for finding narrower ones in any data matrix of the number of columns no more than 500. When tested on simulated datasets, we found that ARBic outperformed seven existing salient biclustering tools by at least 29% on average for recovery, relevance and ${F}_1$scores. In addition, ARBic outperforms all algorithms on real datasets, and is more robust to noises, bicluster shapes, and dataset types.

## MATERIALS AND METHODS

ARBic consists of two components for identifying narrower biclusters and broader biclusters, respectively. We simply incorporated RecBic (26) as one component of ARBic to extract narrower biclusters. We have shown that RecBic, a column-based strategy, is a highly effective and efficient algorithm for recognizing narrower biclusters. The other component of ARBic is designed using a row-based strategy for detecting broader biclusters, which constitutes the main contribution of this article and the rational of the algorithm is described as follows.

For convenience of discussion, we call a submatrix of a data matrix a genuine seed if it can be extended into an actual bicluster, and a false seed otherwise. Obviously, any submatrix is either genuine or false. Intuitively, if a pair of rows in the data matrix pass through a bicluster, then the pair of rows should have a seed (i.e. 2 × *L* submatrix) that is more significant than do two random rows. We assume that the true biclusters could be found by growing the genuine seeds, while the false seeds would phase out during the growth process. The critical step of our row-based strategy is to find an optimum seed (2 × *L* submatrix with L maximized) for each pair of rows in the data matrix as the optimum seed is genuine if the pair of rows pass through a true bicluster, or false one otherwise. It is clear that trend-preserving biclusters are a generalization of all types of biclusters previously mentioned such as constant, row constant, column constant, shift, scale and shift + scale. It can be easily observed that finding an optimum seed of a pair of rows is equivalent to finding the longest path in a pseudo direct acyclic graph (*pDAG*) which is defined on the pair of rows (Section 2.1 Seed optimization). Although finding the longest path in a directed graph is NP-complete, it can be efficiently solved in this scenario because finding the longest path in the *pDAG* can be trivially transformed to finding the longest path in a *DAG* (Section 2.1 Seed optimization). After seed optimization, ARBic greedily grows the optimum seeds one by one until full-sized biclusters are identified using the same method as used for finding initial seeds in each iteration (Section 2.2 Seed extension without noise).

**Figure 1.**
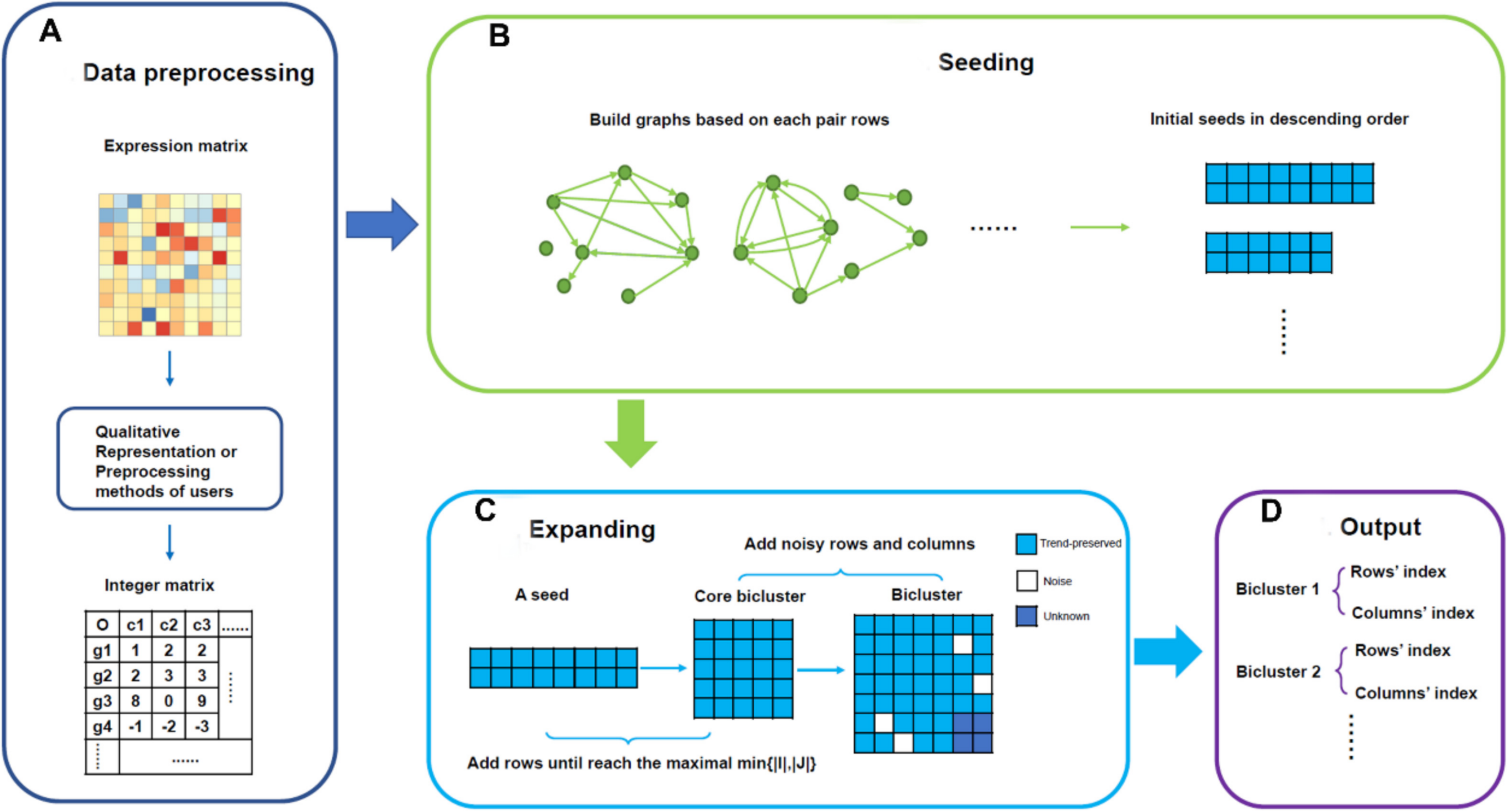
Flowchart of the row-based strategy of ARBic. (**A**) obtains an integer matrix by qualitatively representing the gene expression matrix; (**B**) generates all seeds each is obtained by finding a longest path in the pseudo directed acyclic graph created using a pair of rows of the integer matrix; (**C**) greedily grows the seeds into biclusters to be identified; (**D**) outputs those significant biclusters obtained from (C) in the form (*I, J*), where *I/J* represents the set of row/column indices.

We have previously shown that RecBic works well for any data matrices with the number of columns <500 and the number of rows >tens of thousands. Therefore, ARBic calls RecBic if the data matrix is of column number <500. In addition, ARBic employed as similar data preprocessing step (Figure 1A) as used in QUBIC (see Supplementary Materials for details). Before describing the pseudo codes of ARBic, we elaborate each subroutine of the row-based strategy below.

### Seed optimization

The subroutine finds an optimum seed for each pair of rows of the data matrix by identifying the longest path in the *pDAG* constructed using the pair of rows as follows (Figure 1B).

- Construction of *pDAG*. Given a pair of rows ${r}_i$ and ${r}_j$ in the data matrix, we construct a graph$\ {G}_{ij} = (V,\ E$), where *V* represents the set of vertices corresponding to the column indices *s* of the two rows $( {\begin{array}{@{}*{1}{c}@{}} {{a}_{is}}\\ {{a}_{js}} \end{array}} )$, and E the set of directed edges formed as follows: we add a directed edge (*s, t*) from node s to node *t* (*s ≠ t*) if and only if
  $$\begin{equation*}\left( {\begin{array}{@{}*{1}{c}@{}} {{a}_{is}}\\ {{a}_{js}} \end{array}} \right) \ge \left( {\begin{array}{@{}*{1}{c}@{}} {{a}_{it}}\\ {{a}_{jt}} \end{array}} \right),\ {\rm{i}}.{\rm{e}}.,\ {a}_{is} \ge {a}_{it}{\rm{\ and}}\ {a}_{js} \ge {a}_{jt}.\end{equation*}$$

It follows that an edge (*s, t*) is bidirected if and only if ${a}_{is} = {a}_{it}{\rm{\ and}}\ {a}_{js} = {a}_{jt}.$ Thus, we call ${G}_{ij}$ a *pDAG*.

Now consider the subgraph $G_{ij}^{\prime}$ induced from all bidirected edges of$\ {G}_{ij}$. Clearly, any connected subgraph of $G_{ij}^{\prime}$ is a clique, i.e. a complete subgraph. We denote by $G_{ij}^*$ the graph obtained by contracting each clique of $G_{ij}^{\prime}$ into a single vertex and keeping all directed edges unchanged. Then it follows that $G_{ij}^*$ is acyclic (Supplementary Figures S4–S6).

- Finding the longest directed path in ${G}_{ij}$. Notice that the longest directed path in ${G}_{ij}$ gives an optimum seed determined by rows ${r}_i$ and ${r}_j$. To find the longest path in ${G}_{ij}$, we treat $G_{ij}^*$ as a vertex weighted graph. For each vertex ${v}^* \in {V}^*$, we assign to ${v}^*$ a weight, denoted by $|{v}^*|$, which represents the size of the clique contracted into ${v}^*$. Clearly, the longest weighted directed path ${P}^* = p_1^*\ \to p_2^* \to \cdots \to p_h^*$ in $G_{ij}^*$, where $p_i^*\ {\rm{is\ constracted\ from\ the\ clique}}\ \{ {{i}_1,{i}_2, \cdots ,{i}_{{k}_i}} \},$ corresponds to the longest directed path$P = {p}_1 \to {p}_2 \to \cdots \to {p}_h$in$\ {G}_{ij},$ where ${p}_i = {i}_1 \to {i}_2 \to \cdots \to {i}_{{k}_i}$(see Supplementary Materials for details). Since $G_{ij}^*$ is acyclic, we can easily find the longest weighted directed path ${P}^*$ in $G_{ij}^*$ which gives the longest directed path *P* in ${G}_{ij}$ by blooming each contracted vertex. Then the longest directed path *P* mirrors an optimum seed (i.e. 2 × |*P*| submatrix) which is located in rows ${r}_i$ and ${r}_j$.

### Seed extension without noise

Prior to extending a seed, we need to define an auxiliary seed based on the longest path${\rm{\ }}{P}^* = p_1^*\ \to p_2^* \to \cdots \to p_h^*$ in${\rm{\ }}G_{ij}^*,$ where $p_i^* = \{ {{i}_1,{i}_2, \cdots ,{i}_{{k}_i}} \},\ 1 \le i \le h,$ is a vertex obtained by contracting a clique of vertices ${i}_1,{i}_2, \cdots ,{i}_{{k}_i}$ in ${G}_{ij}$. To do so, we define a mapping $\varphi :\ \{ {1,\ 2, \ldots ,\ n} \}\ \to \{ {1,\ 2, \ldots ,h,\ N} \}$, where $n = | V |,$ such that $\varphi ( j ) = i,\ \forall \ j \in p_i^*,\ i\ = \ 1,\ 2,\ \ldots ,\ h;\ \varphi ( j ) = N,\ \forall \ j \in \{ {1,\ 2, \ldots ,\ n} \}\backslash \cup_{i\ = \ 1}^hp_i^*.$

Then the auxiliary seed is defined as a numerical vector $v\ = \ ( {{v}_1,{v}_2, \cdots ,{v}_n} )$ with its components ${v}_j$ defined as follows:

$$\begin{equation*}{v}_j = i\ {\rm{if\ and\ only\ if\ }}\varphi \left( j \right) = i,{\rm{\ and\ }}{v}_j = D\ {\rm{if\ and\ only\ if\ }}\varphi \left( j \right) = N.\end{equation*}$$

We then restrict the extension to the columns *j* with ${v}_j \ne D$. We greedily recruit a new row into the current bicluster (seed) by finding the longest path in the pDAG constructed using the auxiliary seed and the new row in the same way as used in the seed optimization subroutine (Figure 1C). We repeat the procedure until the current bicluster $B\ = \ \{ {I,J} \}$ has min$\{ {| I |,| J |} \}$ maximized (25), where $I$ and $J$ are the sets of row indices and column indices of the current bicluster (seed), respectively. We call the final $B\ = \ \{ {I,J} \}$ a core bicluster.

### Seed extension with noises

To make ARBic tolerate noises in a data matrix, we extend a core bicluster by adding new rows and columns to it with certain entries violating the trend-preserving pattern (Figure 1C). Let $B\ = {\rm{\ }}\{ {I,J} \}$ be a core bicluster obtained in Section 2.3. For a column $j \notin J$, by ${c}_j$ we denote the proportion of rows in $I$ which are trend-preserved under the condition $J\cup \{ j \}$. For a row $i \notin I$, by ${c}_i$ we denote the proportion of columns in *i* whose trend is the same as the rows in *I* under the condition *J*. We extend the core bicluster *B* by adding to it all the new rows *i* with ${c}_i >\alpha ,$ and all the new columns j with ${c}_j >\alpha$. By ${\rm{default}},{\rm{\ we\ set}}\ \alpha \ = \ 0.9.$

### Scoring function of biclusters

Given a $t \times s$ bicluster in an $m \times n$ data matrix, the probability that it is trend-preserved by chance is ${( {2/{\rm{s}}!} )}^{\rm{t}}$ (14,18). Based on experiments on both simulated and real data, we found that the significance of a trend-preserving $t \times s$ bicluster can be better measured using the function ${( {2nm/s!} )}^t$. Biologically, the more conditions under which $s$ genes are co-expressed, the more highly correlated the genes are. Therefore, to identify broader biclusters using the row-based strategy, we define the score of a $t \times s$ bicluster as:

$$\begin{equation*}BS\ = \ - \log {(\frac{{2nmt}}{{s!}})}^{t}\ \end{equation*}$$

The optimum seed obtained via the seed optimization subroutine should be genuine if both rows *r_i_* and *r_j_* pass through a true bicluster, and false otherwise. Ben-Dor *et al.* (18) developed a technique to estimate the minimum number of rows that a true bicluster should have based on requirements about the significance of the biclusters. Since each pair of rows in a true bicluster are equally capable of providing a genuine seed, it is sufficient to guarantee that at least one pair of rows in each true bicluster to have an opportunity to be selected as the seed by the subroutine of seed optimization. Therefore, we divide the rows in the data matrix into subsets such that each true bicluster has at least one pair of rows belonging to one of the subsets, where the number of subsets can be determined using an estimation formula from (18). Thus, it is sufficient to enumerate all pairs of rows in each subset separately. After seeding, we sort all the seed candidates in a decreasing order in length. Starting with the longest seed as the current bicluster we extend it by calling the extension subroutine without noises. We repeate this with the next longest seed until we get as two times many core biclusters as we want. We then further extend the core biclusters using the extension subroutine with noises. We finally output all biclusters whose scores rank top *o* with the default value set to 50.

### Pseudocodes of ARBic

**Input:** data matrix ${a}_{ij},\ i\ = \ 1, \ldots ,\ m;j\ = \ 1, \ldots ,n$. **${a}_{ij}\ {\rm{represents}}$ preprocessed expression value of gene *i* under condition *j*.

**Output:** biclusters $( {{I}_i,\ {J}_i} ),\ i\ = \ 1, \ldots ,o.$ **${I}_i\ {\rm{and\ }}{J}_i$ are respectively the sets of row indices and column indices of the *i-th* bicluster output, respectively.

**While** $n \le N$ (*N* is set to 500 as default value), call RecBic; **otherwise**

Step 1. Partition rows: partition the $m$ rows of the data matrix ${A}_{m \times n}$ into $k$ subsets, where $k$ is obtained by using the method described in (18) based on *p-*value (by default, *P* = 0.05) of the biclusters to be identified (Supplementary Figure S3).

**P-value of a bicluster is the probability that the bicluster is generated by chance.**

Step 2. Generate seeds: For each of the subsets of rows, we identify all the optimum seeds for each pair of rows in the subset of rows using the subroutine described in Section 2.1. We then sort the seeds in the descending order of their lengths in a list *L*.

Step 3. Extend a seed without noise: We start with the first seed in L as the current trend-preserving bicluster, and repeatedly extend it using the subroutine described in Section 2.2 to obtain a core bicluster.

Step 4. Extend a seed with noises: We extend the core bicluster by adding as many rows and columns as possible using the subroutine described in Section 2.3 with a preset error rate α.

Step 5 Output: We output the resulting bicluster as a candidate, and then remove from the list *L* the seeds with their row pairs in the discovered bicluster. We repeat step 3 until either L is empty or the prespecified number (by default, *o* = 50) of biclusters has been reached.

### Time complexity of ARBic

We now estimate the computational complexity of the ARBic. The number of seeds enumerated by the ARBic is upper bounded by $O( {{m}^2/k} )$. The time consumed to construct a pDAG and to find a longest path in the DAG is $O( {{q}^2{n}^2} ),$ where $q$ is the parameter used QUBIC (25). The time consumed to extend the seeds into final biclusters is $O( {{q}^2{n}^2{m}^2} )$. Therefore, the complexity of ARBic is upper bounded by$\ O( {{q}^2{m}^2{n}^2} )$, where *q* << 1, and $m\ {\rm{and}}\ n$ are the numbers of rows and columns of the data matrix, respectively.

### Evaluation criterion on simulated data

Since there is no golden benchmark real datasets for validating biclustering algorithms, it is a common practice to test them on simulated datasets using two metrics, i.e. recovery and relevance scores, introduced by Prelić *et al.* (27), based on the match scores (Jaccard coefficients) (27) between the predicted biclusters and true ones. Specifically, for two biclusters *b*_1_ and *b*_2_, the match score between them is defined as,

$$\begin{equation*}ms\left( {{b}_1,\ {b}_2} \right) = \frac{{\left| {{b}_1 \cap {b}_2\ } \right|}}{{\left| {{b}_1\cup{b}_2\ } \right|}},\end{equation*}$$

which measures the similarity between the two biclusters, where $| {{b}_1 \cap {b}_2\ } |(resp.\ | {{b}_1\cup{b}_2\ } |$) is the number of data elements in their intersection (*resp*. union). For two sets of biclusters *M_1_* and *M_2_*, the match score between them is defined as below (27),

$$\begin{equation*}s\left( {{M}_1,{M}_2} \right) = \frac{1}{{\left| {{M}_1} \right|}}\mathop \sum \limits_{{b}_1 \in {M}_1} \mathop {\max }\limits_{{b}_2 \in {M}_2} ms\left( {{b}_1,{b}_2} \right),\end{equation*}$$

which measures the average similarity between biclusters in *M_1_* and *M_2_*. Let *T* and *P* be the sets of true and predicted biclusters, respectively, then we call *s*(*T, P*) and *s*(*P, T*) the recovery and relevance score, respectively.

### Evaluation criterion on real expression data

Since the true biclusters in the real datasets are unknown, we evaluated each bicluster identified by each tool using the KEGG biological pathway enrichment method (28), with a Benjamini–Hochberg adjusted *P-*value* < *0.05. A bicluster is enriched if and only if it is enriched for at least one pathway in the KEGG database at $P \le {\rm{\ }}0.05$ after Benjamini-Hochberg multiple-test corrections. If an algorithm outputs *k* biclusters in which $l( { \le {\rm{k}}} )$ are enriched, we define the *biclusters enrichment score* (*F*-score for short) as the proportion of the enriched biclusters i.e.

$$\begin{equation*}F = \frac{l}{k} \times \ 100\% .\end{equation*}$$

For a bicluster *B* = (*I, J*) output by the algorithm, we define the *genes enrichment score*, denoted by *s*, of *B* as the proportion of genes in *B* that are enriched, i.e.

$$\begin{equation*}s = \frac{{\left| {\left( {\mathop \sum \nolimits_{i = 1}^a {K}_i} \right) \cap I} \right|}}{{\left| I \right|}},\end{equation*}$$

where$\ {K}_i,\ i\ = \ 1,2, \ldots ,a$ are enriched pathways by *B* in the KEGG database. Suppose the algorithm outputs *k* biclusters, $\ {B}_i,\ i\ = \ 1,2, \ldots ,$k, of gene enrichment score ${s}_i,i\ = \ 1,\ 2, \ldots ,k$, respectively, then the genes enrichment score of the algorithm (*G-*score for short), is defined as below,

$$\begin{equation*}G = \frac{1}{k}\mathop \sum \limits_{i\ = \ 1}^k {s}_i \times \ 100\% .\end{equation*}$$

Clearly, a good algorithm should enhance *F-* and *G-*scores, simultaneously. To do so, we introduce a new score metric, denoted by ${F}_1$, defined by,

$$\begin{equation*}{F}_1 = \frac{{2\left( {F \times G} \right)}}{{F + G}},\end{equation*}$$

which is the harmonic mean of biclusters enrichment score *F* and genes enrichment score *G*.

## RESULTS

To evaluate ARBic, we compared it with seven currently popular biclustering algorithms, including CPB (10), FABIA (22), ISA (23), OPSM (18), QUBIC2 (19), EBIC (14) and UniBic (16) on various simulated datasets as well as real datasets. These algorithms were chosen because they have been proved to be the top algorithms for finding biclusters in previous studies. We ran all the algorithms with the parameters suggested in their publications on different types of simulated datasets with biclusters of different shapes and patterns, and on five real datasets collected from (29). We restricted our analysis to the row-based strategy on the relatively broad datasets since we have detailed our column-based strategy RecBic earlier (26), and showed that RecBic performed almost perfectly when the data matrix was relatively narrow (26) (much more rows than columns), although it could be collapsed as the data matrix goes broader.

### Generation of simulated datasets

We first generated a data matrix of size 600 × 600. The elements of the data matrix were randomly generated from the standard normal distribution of *N* (0,1). We then randomly selected a predetermined number of submatrices of prespecified numbers of rows and columns, in the data matrix, and rearranged the elements of each submatrix such that all the rearranged submatrices are of prespecified properties. A trend-preserving bicluster was generated by fixing one row in the selected submatrix and rearranging the elements of other rows in the submatrix according to the trend of the fixed row. To better mimic the real data, we allow some elements in each row being equal, i.e. the number of different values in a row may be less than n, the number of columns of the data matrix. In our experiments, we generated three artificial datasets. (i) Datasets implanted with complex trend-preserving biclusters; (ii) datasets implanted with overlapping biclusters; (iii) datasets implanted with noisy biclusters. Note that no noise is introduced in datasets (i) and (ii), and no overlapping biclusters are implanted in datasets (i) and (iii). Since most of the biclustering algorithms compared in this article are not designed to find order-preserving biclusters, we also evaluated the algorithms on datasets implanted with six other types of biclusters (Supplementary Figure S7). We further evaluated them on the artificial data simulated by the tool seqgendiff (30) which was developed especially for simulating RNA-seq data. It can be seen from Supplementary Figure S8 that ARBic still substantially outperforms other algorithms under comparison.

### ARBic almost perfectly identifies trend-preserving biclusters

**Figure 2.**
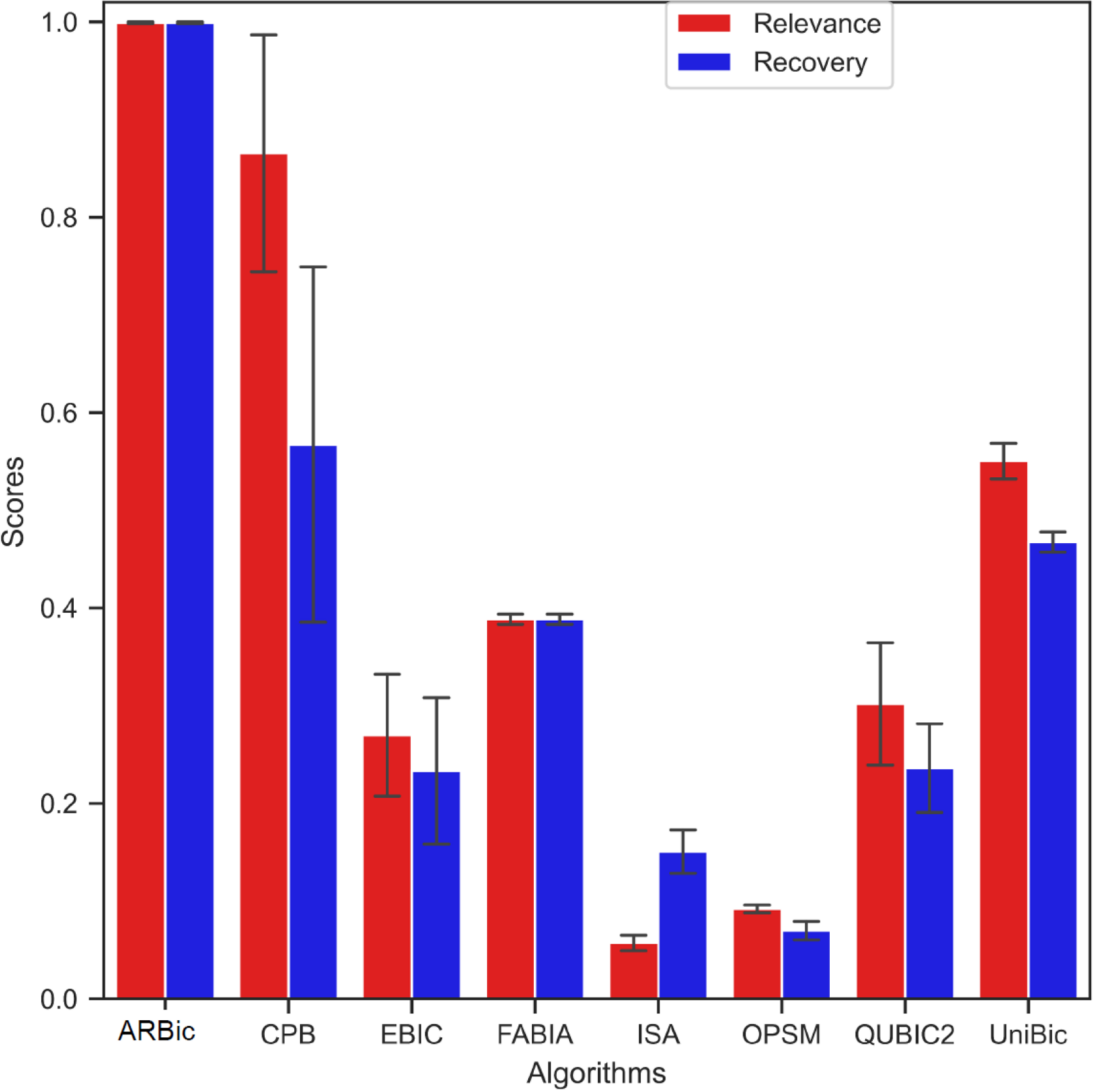
Performance comparisons of the eight algorithms on datasets implanted with six trend-preserving biclusters measured by relevance and recovery scores. The error bars indicate standard errors.

We tested the eight algorithms on the simulated datasets implanted with six trend-preserving biclusters of size 100 × 100, and repeated the experiment four times to reduce variation. As shown in Figure 2, ARBic substantially outperformed the other algorithms with both mean relevance and recovery scores of 0.99. CPB ranks the second-best with mean relevance and recovery scores of 0.9 and 0.6, respectively. UniBic hovers its mean relevance and recovery scores around 0.55 and 0.48, respectively. Although EBIC is comparable to ARBic in finding narrower biclusters (14,26), it collapses as the number of columns increases because it needs more iterations to get rid of local optimum solutions. FABIA performs stably with both mean relevance and recovery scores of 0.4. ARBic also shows a leading advantage in datasets implanted with various biclustering patterns (Supplementary Figure S7). As shown in Supplementary Figure S7, ARBic always reaches to the ceiling value 1 in both mean relevance and recovery scores, and only UniBic is competitive with the ARBic on all five datasets except the one implanted with trend-preserving biclusters. That ARBic improves UniBic on the dataset implanted with trend-preserving biclusters is illustrated in Supplementary Figure S2 where the whole matrix itself is trend-preserved while UniBic outputs bicluster(s) either {{1, 2}, {1, 3, 4, 5}} or {{1, 2}, {1, 2, 4, 5}}. QUBIC2 reaches both mean relevance and recovery scores of 0.95 in the cases where the biclusters are row constant or column constant (Supplementary Figure S7B, C). CPB has its mean relevance scores relatively higher than its mean recovery scores (Supplementary Figure S7A–F). FABIA performs relatively better on trend-preserving, shift, and shift + scale datasets (Supplementary Figure S7A, E, F). EBIC is competitive with ARBic on all the six datasets (Supplementary Figure S7).

### ARBic almost perfectly identifies overlapping biclusters

**Figure 3.**
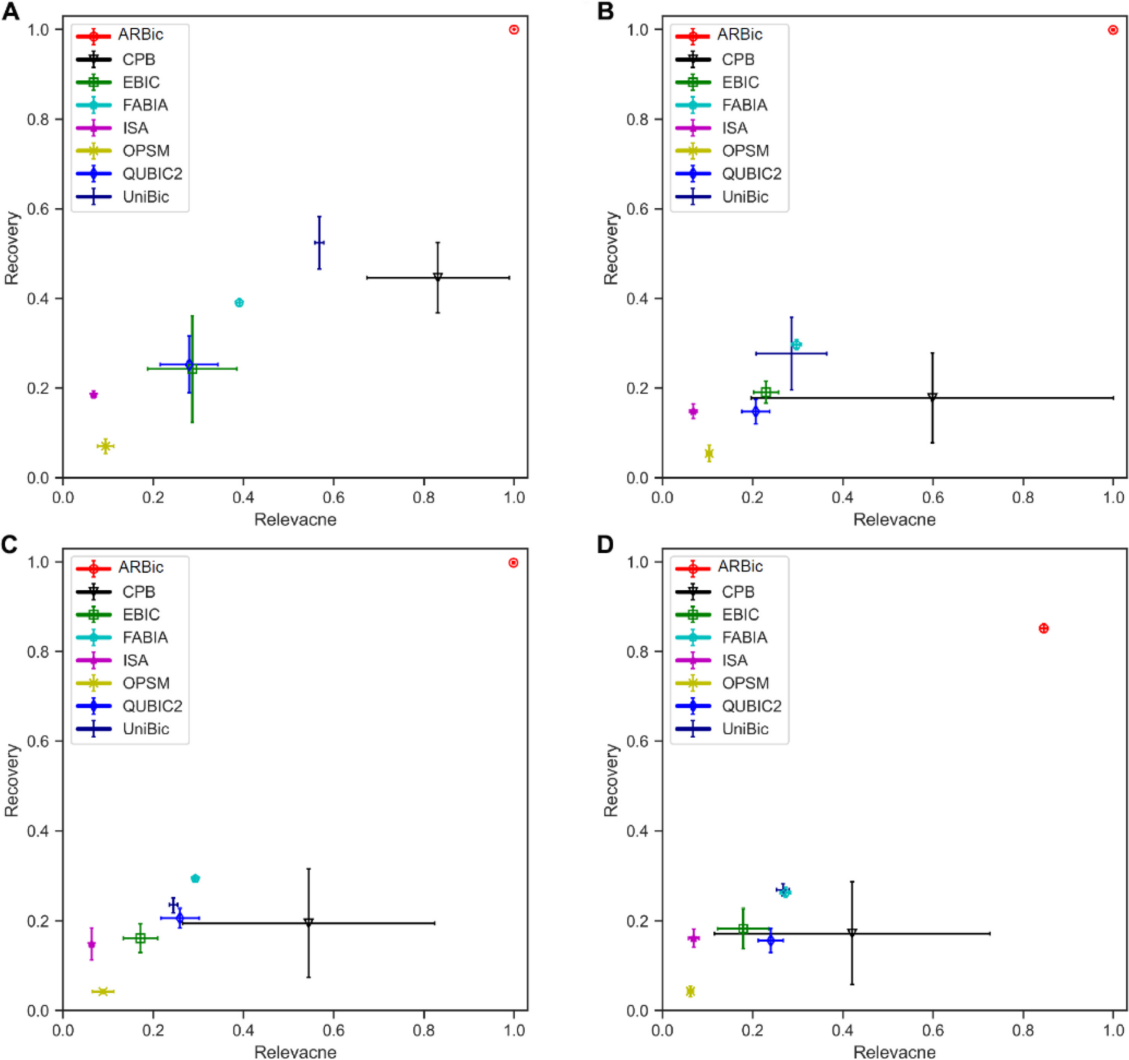
Relationship between relevance and recovery scores for the eight algorithms on datasets containing biclusters with different overlap levels. (**A**) Results on the dataset with no overlapping biclusters. (**B**) Results on the dataset with biclusters with an overlapping level of 30 × 30. (**C**) Results on the dataset with biclusters with an overlapping level of 40 × 40. (**D**) Results on the dataset with the biclusters with an overlapping level of 50 × 50.

Gene regulation is highly combinatorial and gene products can participate in multiple pathways, which means that overlaps between genes of biclusters in expression data matrices are widespread. We compared the algorithms for identifying overlapping biclusters on four simulated datasets implanted with six overlapping 100 × 100 biclusters in a 600 × 600 data matrix having four overlapping levels: 0 × 0 (no overlap), 30 × 30, 40 × 40 and 50 × 50, where overlapping level $s \times t$ represents two biclusters having s rows and t columns in common. To ensure the overlapping biclusters to be trend-preserved, we set the elements in the overlapping region to a constant value. As shown in Figure 3, the higher the overlapping level, the harder the algorithms extract true biclusters. QUBIC2 performs stably, but it has poor relevance and recovery scores. The relevance score of CPB and the recovery score of UniBic decrease sharply, and the performaces of both FABIA and EBIC decrease as the overlapping level goes up. ARBic performs almost perfectly until overlapping level is beyond 50${\rm{\ }} \times$ 50, but it remains the priority to the others. Concretely, ARBic achieves on an average 0.99 in both relevance and recovery scores on datasets with overlapping levels of 0 × 0, 30 × 30, 40 × 40, respectively, and goes down with the overlapping level beyond 50 × 50.

### ARBic is robust to noise

**Figure 4.**
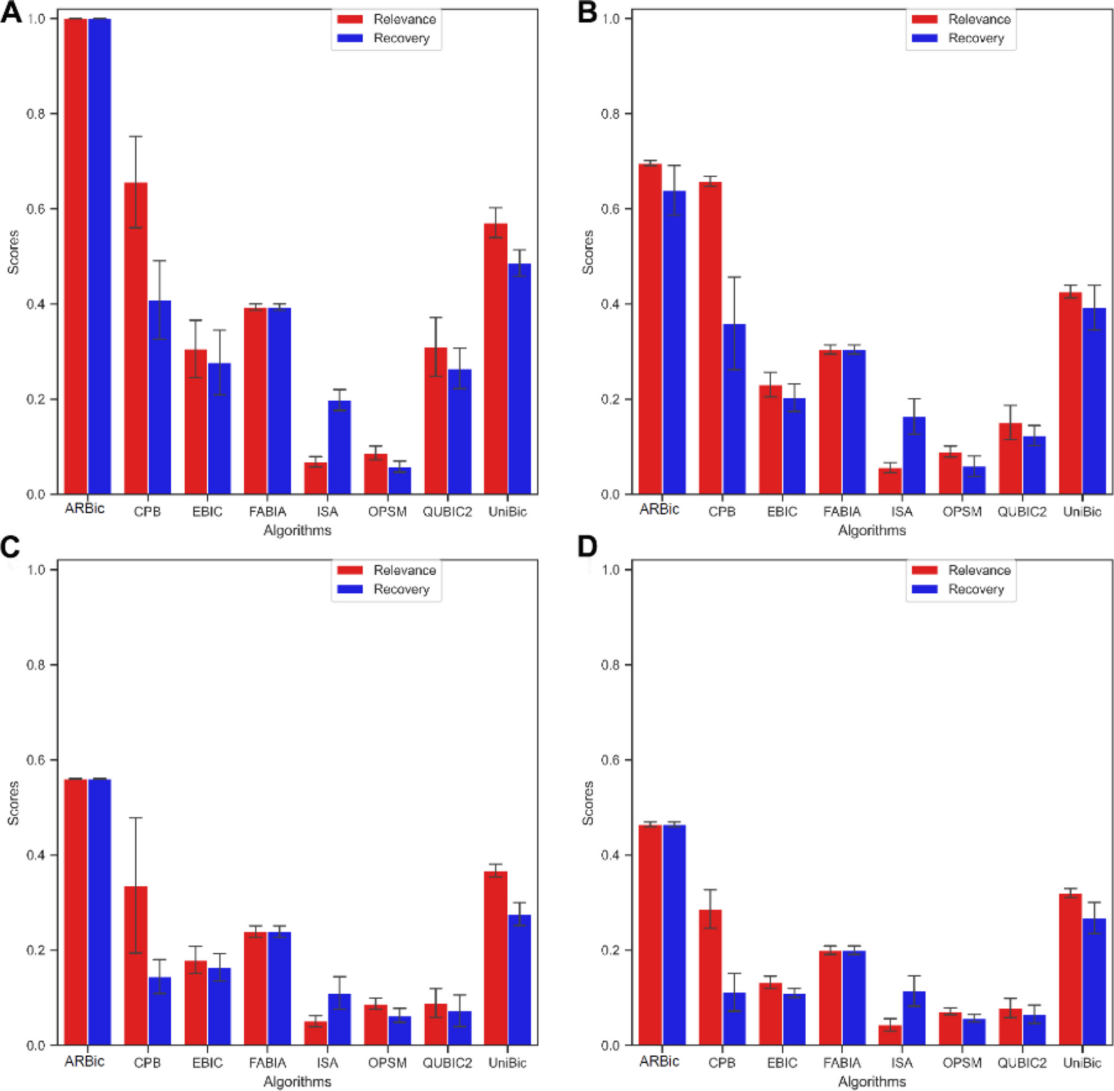
Performance comparison of the eight algorithms on datasets of different noise levels measured by relevance and recovery scores. (**A**) Results on the dataset with no noisy biclusters. (**B**) Results on the dataset with the biclusters noise level of 0.1. (**C**) Results on the dataset with the biclusters noise level of 0.2. (**D**) Results on the dataset with the biclusters noise level of 0.3. The error bars indicate standard errors.

We define the noise level in a bicluster to be the maximum of row noise levels in the bicluster, where the row noise level is the ratio of the number of elements whose removal will make the trend-preserving row to the consensus of the bicluster over the number of all elements in the row. We evaluated the impact of noise levels on the performance of the eight tools on datasets containing three biclusters of size 100 × 100 with different noise levels (0.0, 0.1, 0.2 and 0.3) in a data matrix of size 600 × 600 (see Materials and Methods). As shown in Figure 4A–D, the performaces of all the compared algorithms decrease as the noise level grows up, which is in line with our expectation. In contrast, FABIA performs stably due to its well-modeled data noise parameter. UniBic, the runner-up overall shows competitive performance as the noise level grows up. ARBic consistently achieves the highest relevance and recovery scores on all the datasets, implying that it has the highest tolerance to noise among the eight tools.

### Performance of eight tools on real datasets

Since RecBic performs well on the simulated data matrices with a relatively small number of columns/conditions, we compared ARBic with other algorithms on five real datasets with a larger number (>500) of columns (details are in Supplementary Materials). These datasets include two from *E. coli*, two from yeast, and one from human tissues, which have been used in (29) (Supplementary Table S1). In order to minimize the deviation of selecting parameters of tools, we ran more than 20 times (Supplementary Table S2) using different parameters each time for each method, and used the median score as the evaluation criterion. Since OPSM cannot get results in an acceptable time on the human dataset, both scores of OPSM on this data are set to 0.

As shown in Figure 6, ARBic reaches the highest median ${F}_1$-score of 0.64 overall, followed by QUBIC2 and UniBic with ${F}_1$-scores of 0.34 and 0.35, respectively. On the *E. coli* Colombos and *E. coli* Dream5 datasets, ARBic substantially outperforms other algorithms. Visualization of two biclusters found by ARBic on the E.coli Dream5 dataset is shown in Supplementary Figure S9. All algorithms sharply decreased in performance over the human dataset, while ARBic still keeps the best ${F}_1$-score of 0.25. On the Yeast Dream5 dataset, UniBic achieves the best ${F}_1$-score of 0.72, followed by ARBic with an ${F}_1$-score of 0.68. EBIC, ISA and FABIA, however, fluctuate widely across different datasets.

**Figure 5.**
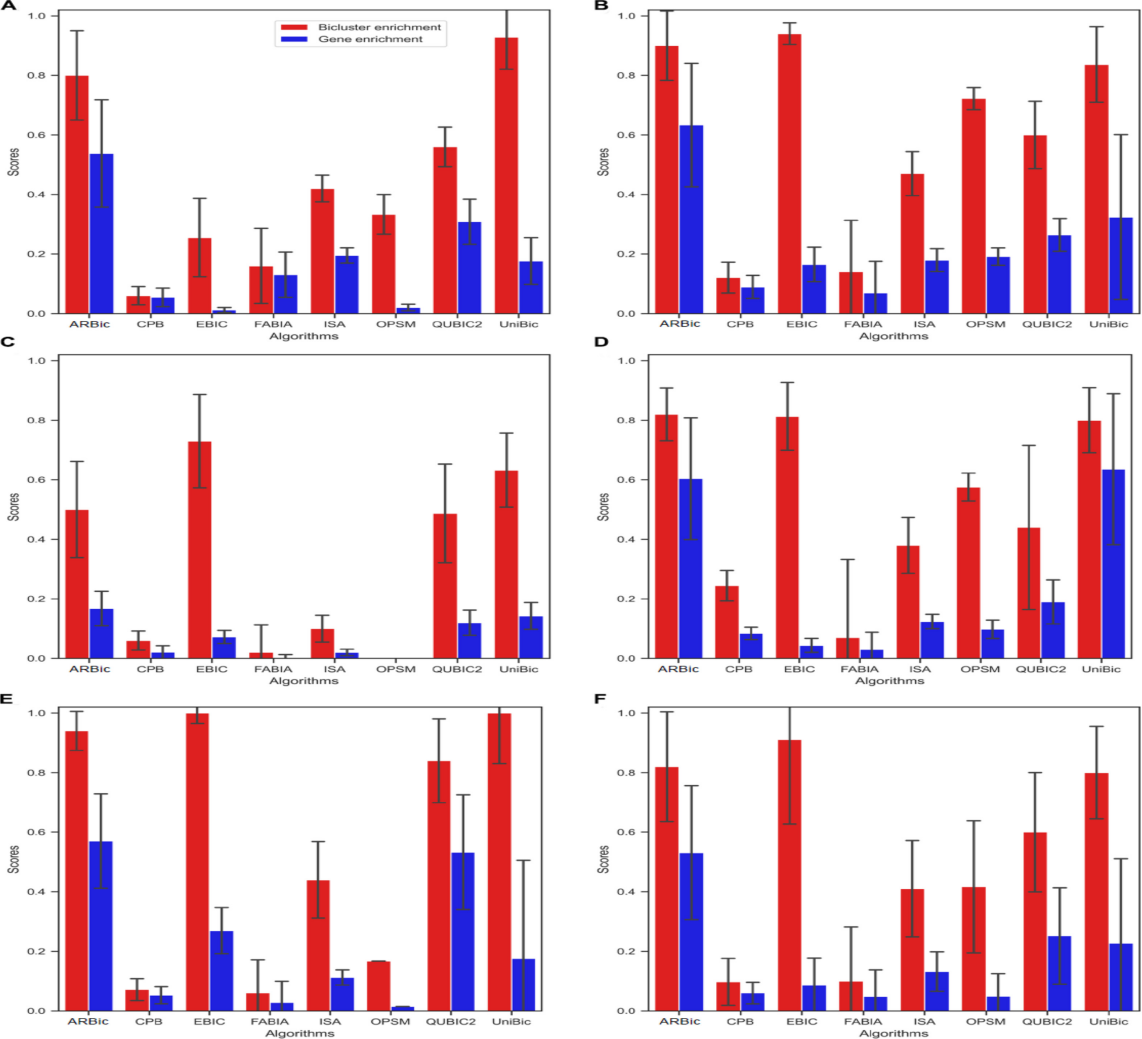
Performance comparisons of the eight algorithms on five real datasets measured by *F-* and *G-*scores. (**A**) *E. coli* COLOMBOS dataset; (**B**) *E. coli* Dream5 dataset; (**C**) human seek GPL5175 dataset; (**D**) yeast Dream5 dataset; (**E**) yeast GPL2529 dataset; (**F**) the average on the five datasets.

As shown in Figure 5, ARBic reaches the best *G*-score of 0.53 and the second highest *F-*score of 0.84 overall. Other algorithms have their own advantages. EBIC achieves the highest *F*-score of 0.91 overall, but its median *G-*score is merely 0.09. EBIC tends to find more genes, which makes the biclusters it finds easier to be enriched, while leading to numerous false-positive genes. UniBic achieves a similar *F*-score to ARBic. QUBIC2 and UniBic are ranked suboptimum in their *G*-scores. QUBIC2 achieves the second-best *G*-score of 0.25. The reason for the sharp decline in the performance of all algorithms on human datasets is that humans have more complicated pathways than do *E. coli* and yeast.

**Figure 6.**
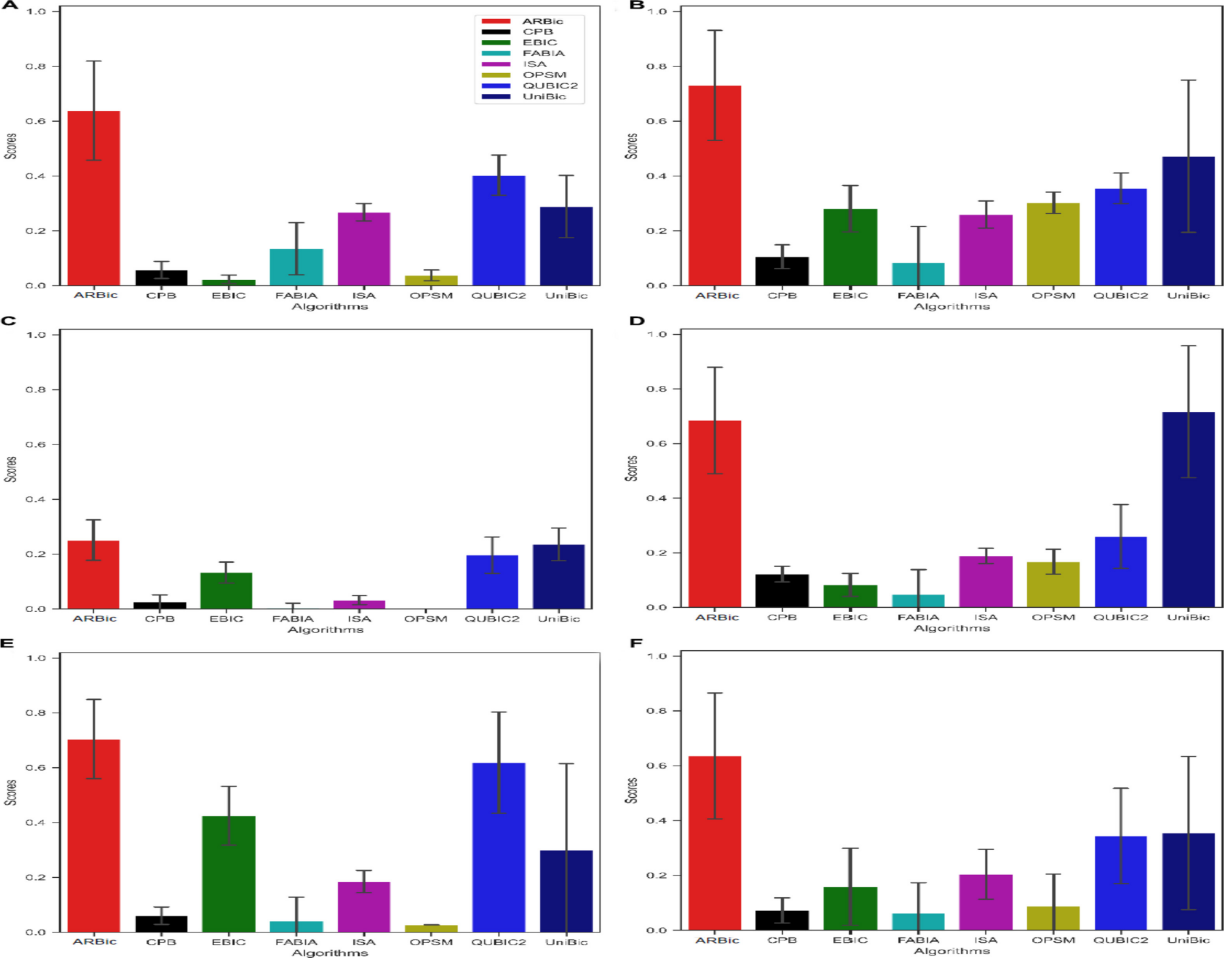
Performance comparison of the eight algorithms on five real datasets in terms of ${F}_1$-scores. (**A**) *E. coli* COLOMBOS dataset; (**B**) *E. coli* Dream5 dataset; (**C**) human seek gpl5175 dataset; (**D**) yeast Dream5 dataset; (**E**) yeast GPL2529 dataset; (**F**) the average on the five datasets.

**Figure 7.**
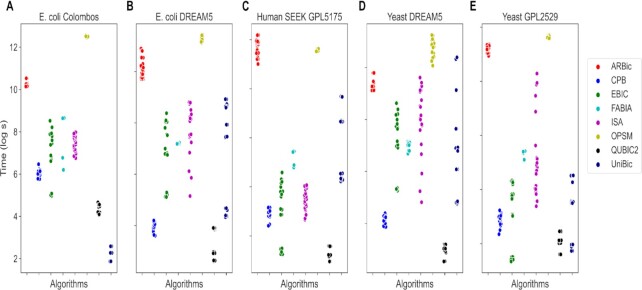
Comparison of the running times of eight algorithms on five real datasets. The base of the logarithmic function is 2.

Each combination of possible parameters in Supplementary Table S2 is associated with a data point in each chart of Figure 7. Since EBIC is a genetic algorithm, the time cost of EBIC varies greatly with the number of iterations. Using the quantile method, UniBic and QUBIC2 only need to deal with up-/down-regulated genes, not neutral genes, causing them to speed up substantially. The ARBic is more time consuming than most of others, but it is acceptable in practice. The average running time of the compared algorithms is summarized in Supplementary Table S3.

## DISCUSSION

Ideally, a biclustering algorithm should be able to identify both narrower and broader biclusters in a data matrix. To our best knowledge, ARBic is the first such an algorithm with such capability. ARBic achieves this goal by integrating our earlier RecBic algorithm targeting narrower biclusters, and new graph-theoretic algorithm targeting broader ones. Unlike RecBic with time complexity of *O*($m{n}^3$), which cannot run on the even intermediately broad data matrix, the new algorithm with time complexity of *O(*${q}^2{m}^2{n}^2$*)*, is able to run on quite broad data matrix. The algorithm finds a bicluster based on a seed associated with the longest path on a pDAG. Therefore, the seed is globally optimized, the first of its kind to our best knowledge, which accurately uses graphs to model trend-preserving biclusters, so that ARBic compensates for the drawback of UniBic that locates a seed by identifying the longest common subsequence between two sequences. Moreover, using a new objective function, the algorithm is able to more accurately discriminate the functionally related genes. Comparing the algorithm with other seven salient biclustering algorithms, we show that our algorithm substantially and consistently outperforms all these algorithms in identifying various kinds of biclusters in simulated datasets as well as functional biclusters in real gene expression datasets. Not only can our algorithm finds more functionally related modules, but also the biclusters we obtained have more enriched genes. Therefore, ARBic could be very useful to analyze gene expression data and find co-regulated genes, and thus could help infer underlying gene regulatory networks.

As the cost of sequencing decreases, there will be more and more gene expression data generated with higher column dimensions. Although the ARBic has been highly efficient, it will consume numerous times when dealing with data of this scale. However, as ARBic can be easily parallelized as the seeds could be separately and parallelly amplified into the final biclusters, it can be adapted for clustering larger RNA-seq data.

## CONCLUSIONS

Biclustering has been widely used in analyzing gene expression data as it provides flexibility to identify co-expressed genes under some but not necessarily all conditions/samples, which the traditional clustering methods lack. However, all the existing biclustering algorithms were designed to identify a special kind of biclusters, broader or narrower but not both. In this study, by combining our earlier column-based procedure RecBic (26) with a new row-based one, we developed a novel bicluster algorithm ARBic to discover trend-preserving biclusters, be it narrower or broader, in any type of data matrices. To our best knowledge, ARBic is the first to iteratively identify a bicluster with a global optimum in each iteration. Comparing ARBic with seven existing state-of-the-art biclustering algorithms, we showed that RecBic substantially and consistently outperformed all of them in identifying various kinds of biclusters in simulated datasets as well as functionally enriched biclusters in real gene expression datasets. ARBic can be a useful tool to identify both narrower and broader biclusters in large gene expression datasets, thereby helps to elucidate transcriptional regulation networks.

## DECLARATIONS

Abbreviations: pDAG, pseudo direct acyclic graph.

Ethics approval and consent to participate: Not applicable.

## DATA AVAILABILITY

Code: https://github.com/holyzews/ARBic. Data: https://doi.org/10.5281/zenodo.5121018.

## Supplementary Material

lqad009_Supplemental_File

## References

[1] Reuter J.A. , Spacek D.V., Snyder M.P. High-throughput sequencing technologies. Mol. Cell. 2015; 58:586–597. 26000844 10.1016/j.molcel.2015.05.004 PMC4494749

[2] Van Dam S. , Vosa U., Der Graaf A.V., Franke L., De Magalhaes J.P. Gene co-expression analysis for functional classification and gene–disease predictions. Brief. Bioinf. 2017; 19:575–592. 10.1093/bib/bbw139 PMC6054162 28077403

[3] Morgan J.N. , Sonquist J.A. Problems in the analysis of survey data, and a proposal. J. Am. Statist. Assoc. 1963; 58:415–434.

[4] Hartigan J.A. Direct clustering of a data matrix. J. Am. Statist. Assoc. 1972; 67:123–129.

[5] Cheng Y. , Church G.M. Intelligent Systems in Molecular Biology. 2000; 8:93–103. 10977070

[6] Bryan K. , Cunningham P. 2006 IEEE Symposium on Computational Intelligence and Bioinformatics and Computational Biology. 2006; IEEE 1–8.

[7] Carmonasaez P. , Pascualmarqui R.D., Tirado F., Carazo J.M., Pascualmontano A. Biclustering of gene expression data by non-smooth non-negative matrix factorization. BMC Bioinf. 2006; 7:78–78. 10.1186/1471-2105-7-78 PMC1434777 16503973

[8] Gu J. , Liu J.S. Bayesian biclustering of gene expression data. BMC Genomics. 2008; 9:1–10. 18366617 10.1186/1471-2164-9-S1-S4 PMC2386069

[9] Henriques R.T. , Ferreira F.L., Madeira S.C. BicPAMS: software for biological data analysis with pattern-based biclustering. BMC Bioinf. 2017; 18:82–82. 10.1186/s12859-017-1493-3 PMC5290636 28153040

[10] Bozdag D. , Parvin J.D., Catalyurek U.V. International Conference on Bioinformatics. 2009; 151–163.

[11] Kung S. , Mak M., Tagkopoulos I Symmetric and asymmetric multi-modality biclustering analysis for microarray data matrix. J. Bioinform. Comput. Biol. 2006; 4:275–298. 16819784 10.1142/s0219720006002065

[12] Li H. , Chen X., Zhang K., Jiang T A general framework for biclustering gene expression data. J. Bioinform. Comput. Biol. 2006; 4:911–993. 17007074 10.1142/s021972000600217x

[13] Reiss D.J. , Baliga N.S., Bonneau R. Integrated biclustering of heterogeneous genome-wide datasets for the inference of global regulatory networks. BMC Bioinf. 2006; 7:280–280. 10.1186/1471-2105-7-280 PMC1502140 16749936

[14] Orzechowski P. , Sipper M., Huang X., Moore J.H. EBIC: an evolutionary-based parallel biclustering algorithm for pattern discovery. Bioinformatics. 2018; 34:3719–3726. 29790909 10.1093/bioinformatics/bty401 PMC6198864

[15] Henriques R. , Madeira S.C. BSig: evaluating the statistical significance of biclustering solutions. Data Mining Knowl. Discov. 2018; 32:124–161.

[16] Wang Z. , Li G., Robinson R.W., Huang X. UniBic: sequential row-based biclustering algorithm for analysis of gene expression data. Sci. Rep. 2016; 6:23466–23466. 27001340 10.1038/srep23466 PMC4802312

[17] Eren K. , Deveci M., Kucuktunc O., Catalyurek U.V. A comparative analysis of biclustering algorithms for gene expression data. Briefings Bioinf. 2013; 14:279–292. 10.1093/bib/bbs032 PMC3659300 22772837

[18] Bendor A. , Chor B., Karp R.M., Yakhini Z. Discovering local structure in gene expression data: the order-preserving submatrix problem. J. Comput. Biol. 2003; 10:373–384. 12935334 10.1089/10665270360688075

[19] Xie J. , Ma A., Zhang Y., Liu B., Cao S., Wang C., Xu J., Zhang C., Ma Q. QUBIC2: a novel and robust biclustering algorithm for analyses and interpretation of large-scale RNA-seq data. Bioinformatics. 2019; 36:1143–1149. 10.1093/bioinformatics/btz692 PMC8215922 31503285

[20] Madeira S.C. , Oliveira A.L. Biclustering algorithms for biological data analysis: a survey. IEEE/ACM Trans. Comput. Biol. Bioinf. 2004; 1:24–45. 10.1109/TCBB.2004.2 17048406

[21] Lazzeroni L. , Owen A. Plaid models for gene expression data. Statistica Sinica. 2002; 12:61–86.

[22] Hochreiter S. , Bodenhofer U., Heusel M., Mayr A., Mitterecker A., Kasim A., Khamiakova T., Van Sanden S., Lin D., Talloen W. FABIA: factor analysis for bicluster acquisition. Bioinformatics. 2010; 26:1520–1527. 20418340 10.1093/bioinformatics/btq227 PMC2881408

[23] Bergmann S. , Ihmels J., Barkai N. Iterative signature algorithm for the analysis of large-scale gene expression data. Phys. Rev. E. 2003; 67:031902. 10.1103/PhysRevE.67.031902 12689096

[24] Murali T.M. , Kasif S. Pacific Symposium on Biocomputing. 2002; 77–88. 12603019

[25] Li G. , Ma Q., Tang H., Paterson A.H., Xu Y. QUBIC: a qualitative biclustering algorithm for analyses of gene expression data. Nucleic Acids Res. 2009; 37:e101. 19509312 10.1093/nar/gkp491 PMC2731891

[26] Liu X. , Li D., Liu J., Su Z., Li G RecBic: a fast and accurate algorithm recognizing trend-preserving biclusters. Bioinformatics. 2020; 36:5054–5060. 32653907 10.1093/bioinformatics/btaa630

[27] Prelić A. , Bleuler S., Zimmermann P., Wille A., Bühlmann P., Gruissem W., Hennig L., Thiele L., Zitzler E. A systematic comparison and evaluation of biclustering methods for gene expression data. Bioinformatics. 2006; 22:1122–1129. 16500941 10.1093/bioinformatics/btl060

[28] Yu G. , Wang L.G., Han Y., He Q.Y. clusterProfiler: an R package for comparing biological themes among gene clusters. Omics. 2012; 16:284–287. 22455463 10.1089/omi.2011.0118 PMC3339379

[29] Saelens W. , Cannoodt R., Saeys Y. A comprehensive evaluation of module detection methods for gene expression data. Nat. Commun. 2018; 9:1090. 29545622 10.1038/s41467-018-03424-4 PMC5854612

[30] Gerard D. Data-based RNA-seq simulations by binomial thinning. BMC Bioinf. 2020; 21:206. 10.1186/s12859-020-3450-9 PMC7245910 32448189

