# Supplementary material for: ARBic: an all-round biclustering algorithm for analyzing gene expression data
Source: NAR Genom Bioinform. 2023 Jan 31;5(1):lqad009. doi: 10.1093/nargab/lqad009 (PMC9887595; doi:10.1093/nargab/lqad009)
Supplement: lqad009_Supplemental_File [file lqad009_supplemental_file.docx]

**Supplementary materials for the manuscript**

**ARBic: a new biclustering algorithm via bicluster seeding optimization**

Xiangyu Liu^1^, Ting Yu^1^, Xiaoyu Zhao^1^, Chaoyi Long^1^, Renmin Han^1^, Zhengchang Su^3^, Guojun Li^1,2,*^

^1^Research Center for Mathematics and Interdisciplinary Sciences, ^2^School of Mathematics, Shandong University, Jinan 250100, China; ^3^School of Mathematical Science, Liaocheng University, Liaocheng 252000, China; ^4^Department of Bioinformatics and Genomics, The University of North Carolina at Charlotte, Charlotte, NC 28223, USA.

**Contents:**

[1. Definitions of bicluster types 2](#_Toc97333314)

[2. Counterexample of UniBic 3](#_Toc97333315)

[3. Find the longest directed path in *G_ij_* 3](#_Toc97333316)

[4. Find an integer k to partition background matrix into subsets of rows and columns 4](#_Toc97333317)

[5. An example of row-based stratey 5](#_Toc97333318)

[6. ARBic outperformed the existing tools for finding biclusters with different patterns 7](#_Toc97333319)

[7. Details of real datasets used in the maintext 8](#_Toc97333320)

[8. Details of parameters used in the main text 8](#_Toc97333321)

[9. Data preprocessing step 9](#_Toc97333322)

[10. Average running time of algorithms 10](#_Toc97333323)

1. **Definitions of bicluster types**

A bicluster is said to be **constant** if all the elements in the bicluster are same, **shift** if each row can be obtained from a base row by adding a number to the base row, **scale** if each row can be obtained from a base row by multiplying the base row by a number, and **scale-shift** if each row can be obtained from a base row by scaling and shifting simultaneously. Obviously, the first three types can be viewed as special cases of scale-shift pattern. A bicluster is said to be **trend-preserved** if there is a column permutation such that each permutated row is monotonic, i.e. increased or decreased (Figure S1 E). Obviously, trend-preserved biclusters are most general and meaningful local structures in gene expression data including scale-shift patterns as special cases. If we by *π_j_* denote the typical value under condition *j*, by $\alpha_{i}$ and $\beta_{i}$represent adjustments used in patterns for each gene *i*, then the elements b_ij_ of a shift-scale bicluster can be written as *b_ij_=α_i_×π_j_+β_i_* (Figure S1 D). Then constant (Figure S1 A) , shift (Figure S1 B) and scale (Figure S1 C) biclusters are all special cases of the scale-shift pattern with specific $\alpha_{i}$ or/and $\beta_{i}$.

|  | *c*_1_ | *c*_2_ | *c*_3_ |
| --- | --- | --- | --- |
| *g*_1_ | 1 | 1 | 1 |
| *g*_2_ | 1 | 1 | 1 |
| *g*_3_ | 1 | 1 | 1 |

|  | *c*_1_ | *c*_2_ | *c*_3_ |
| --- | --- | --- | --- |
| *g*_1_ | 1 | 2 | 3 |
| *g*_2_ | 2 | 3 | 4 |
| *g*_3_ | 3 | 4 | 5 |

|  | *c*_1_ | *c*_2_ | *c*_3_ |
| --- | --- | --- | --- |
| *g*_1_ | 1 | 2 | 3 |
| *g*_2_ | 2 | 4 | 6 |
| *g*_3_ | 3 | 6 | 9 |

**A** Constant **B** Shift **C** Scale

|  | *c*_1_ | *c*_2_ | *c*_3_ |
| --- | --- | --- | --- |
| *g*_1_ | 2 | 3 | 4 |
| *g*_2_ | 3 | 5 | 7 |
| *g*_3_ | 4 | 7 | 10 |

|  | *c*_1_ | *c*_2_ | *c*_3_ |
| --- | --- | --- | --- |
| *g*_1_ | 1 | 7 | 5 |
| *g*_2_ | 2 | 6 | 4 |
| *g*_3_ | -1 | -5 | -2 |

**D** Shift-Scale **E** Trend-Preserved

**Figure S1** Five types of bicluster types. a) Constant bicluster with *π_j_ = 1, j =1, 2, 3; α_i_ = 1, β_i_ = 0,* *i = 1, 2, 3*. b) Shift bicluster with *π_j_ = j* for *j = 1, 2, 3; α_i_ = 1, β_i_ = i - 1* for *i=1, 2, 3.* c) Scale bicluster with *π_j_ =* *j* for *j* = *1, 2, 3; α_i_ = i, β_i_ = 0* for *i = 1, 2, 3.* d) Shift-scale bicluster with *π_j_ = j* for *j* = *1,2,3* and *α_i_ = i, β_i_ = 1* for *i = 1, 2, 3*. **e.** Trend-preserved bicluster with *b_i2_ ≥ b_i3_ ≥ b_i1_* for *i = 1, 2*; *b_32_ ≤ b_33_ ≤ b_31_*.

1. **Counterexample of UniBic**

The biclustering algorithm UniBic we previously developed attempted to discover biclusters of trend-preserved patterns. It applied the Longest Common Subsequence (LCS) finding algorithm to each pair of rows of an index matrix to locate seeds. However, the seed location procedure was not globally optimized. Figure S2 shows a counterexample where the whole bicluster itself is trend-preserved, but then UniBic can only output {{*1, 2*}, {*1, 3, 4, 5*}} or {{*1, 2*}, {*1, 2, 4, 5*}}.

**
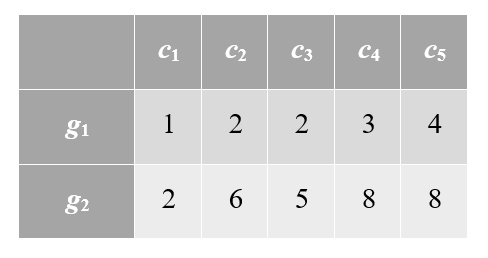
**

**Figure S2** Counterexample of UniBic. The whole bicluster itself is trend-preserved, but UniBic outputs {{*1, 2*}, {*1, 3, 4, 5*}} or {{*1, 2*}, {*1, 2, 4, 5*}}.

1. **Find the longest directed path in *G_ij_***

For a direct graph *G_ij_* defined by two vectors, we find its longest paths by traversing an acyclic graph $G_{ij}^{*}$ obtained by contracting each clique into a node. Firstly, we assign to each node in *G_ij_* a weight of number 1, and contract each clique in *G_ij_* into a node of $G_{ij}^{*}$ with weight being the sum of node weights of the clique. Then we find the path with the maximum weight sum of nodes in directed acyclic graph $G_{ij}^{*}$. At last by blooming the cliques, we get the node set of longest paths in *G_ij_*. We find the path with the maximum weight sum of nodes in directed acyclic graph $G_{ij}^{*}$ by using dynamic programming:

1. Initialize the node weight *w*(*v*) in $G_{ij}^{*}$. The weight of a contracted node is the number of nodes in the clique while the others are all the number 1.
2. Let *V* be the set of all nodes, *S* the set of vertices with indegree 0, and *Ms[v_i_]* the maximum weight among the paths started at nodes in *S* and ended at node *v*_i_ ( *v*_i_∈*V*\*S*)*.* Let M be a set of *Ms[v_i_]*, for all *v*_i_∈*V*\*S*. Let *Mp[v_i_]* represent the parent node of *v*_i_ defined by the path ended in *v*_i_ of weight *Ms[v_i_]*.
3. For each *v_j_∈V\S* in Topological Sorting order (a topological sorting order of a directed graph is a linear ordering of its vertices such that for every directed edge *v*_i_*v*_j_ from vertex *v*_i_ to vertex *v*_j_*, v*_i_ comes before *v*_j_ in the ordering) do:

*Ms[v_j_]=max{Ms[v_i_]+w(v_j_):* *v_i_* is a parent node of *v*_j_}, where *w*(*v*_j_) is the weight of *v*_j_. Set *Mp[v_j_]=v_i_* if *v*_i_ = arg *Ms[v_j_]*.

1. Find the maximum value *Ms[v_t_]* in M*.* The desired path in$G_{ij}^{*}$can be traced back through *Mp[v_t_]*. Then the longest path in *G_ij_* can be obtained through blooming the cliques in the desired path.
2. **Find an integer k to partition background matrix into subsets of rows and columns**

A bicluster is thought to be a significant bicluster, if it can’t be simply random generated by background matrix . For a background matrix $A_{n\times m}$, the probability that A has an order-preserved submatrix of size *t×s* is estimated by the technique established in [1]. For a randomly selected row, the probability that the entries in its s columns support rightly or reversely a given column index permutation is *2/s*!. As each row is independent with each other, and there are *m···(m-s+1)* ways to choose a complete model of size s, the probability *P* of having *t* rows supporting the column index permutation is estimated as:

$P=\left\{ \begin{aligned} U,if U<1 \\ 1, if U\geq1 \end{aligned} \right.$ , $U=m\cdots(m-s+1)C_{n}^{t}{(\frac{2}{s!})}^{t}{(1-\frac{2}{s!})}^{n-t}$

| Size\s | 6 | 7 | 8 | 9 | 10 | 11 | 12 | 13 | 14 | 15 | 16 |
| --- | --- | --- | --- | --- | --- | --- | --- | --- | --- | --- | --- |
| n=1000, m=50 | 21 | 12 | 8 | 6 | 5 | 5 | 5 | 4 | 4 | 3 | 3 |
| n=5000, m=50 | 47 | 20 | 12 | 8 | 6 | 5 | 5 | 4 | 4 | 4 | 3 |
| n=10000, m=50 | 71 | 27 | 14 | 9 | 7 | 6 | 5 | 4 | 4 | 4 | 3 |
| n=15000, m=50 | 93 | 32 | 16 | 10 | 7 | 6 | 5 | 4 | 4 | 4 | 4 |
| n=1000, m=100 | 23 | 13 | 9 | 7 | 6 | 5 | 4 | 4 | 4 | 4 | 4 |
| n=5000, m=100 | 50 | 22 | 13 | 9 | 7 | 6 | 5 | 5 | 4 | 4 | 4 |
| n=10000, m=100 | 76 | 29 | 16 | 10 | 8 | 7 | 6 | 5 | 5 | 4 | 4 |
| n=15000, m=100 | 98 | 35 | 18 | 11 | 8 | 7 | 6 | 5 | 5 | 4 | 4 |

**Figure S3** The value of row number t for different column number s when U < 0.05.

From the Figure S3 we can see that in real data, when the input matrices are of about 10,000 rows, *k=t= 4* would be sufficient for us to anchor a seed for row-based strategy.

Because the column-based strategy aims to identify very narrow biclusters, we set a small *k* (*k=t=3*) for this strategy to anchor seeds. We don’t select *k=t=2*, because it’s a trivail condition.

1. **An example of row-based stratey**

The input matrix is shown in Figure S4. We choose a pair of rows (*i, j*) as the initial seed. Following the rules in main text, we construct graph *G_ij_* based on the rows i and j. Then we contract the clique consisting of nodes *e* and *d* into a new node *g,* forming the graph *G* ij* (see FigureS5). We found the longest path(*g→b→c→a*) in *G* ij*by applying the dynamic programming algorithm on the graph *G* ij*, and a longest path (*e→d→b→c→a* or *d→e→b→c→a*) by blooming the clique, and thus a seed ({*i,j*}, {*a, b, c, d, e*}) in *G_ij_*. We then add the row *k* to the current seed to get the next seed (bicluster) of one more row by constructing a graph *G_ijk_* with nodes *a*, *b*, *c*, *d*, *e* based on the rows *i* and *k* using the same way as used in constructing *G_ij_* (see FigureS6). The longest path *e→d→b→c* in *G_ijk_* provides the trend-preserved bicluster {{*i,j,k*},{*b,c,d,e*}}.

|  | *a* | *b* | *c* | *d* | *e* | *f* |
| --- | --- | --- | --- | --- | --- | --- |
| *I* | 1 | 2 | 2 | 3 | 3 | 0 |
| *J* | 2 | 6 | 5 | 8 | 8 | 7 |
| *K* | 10 | 7 | 4 | 8 | 11 | 1 |

**Figure S4** Input matrix.


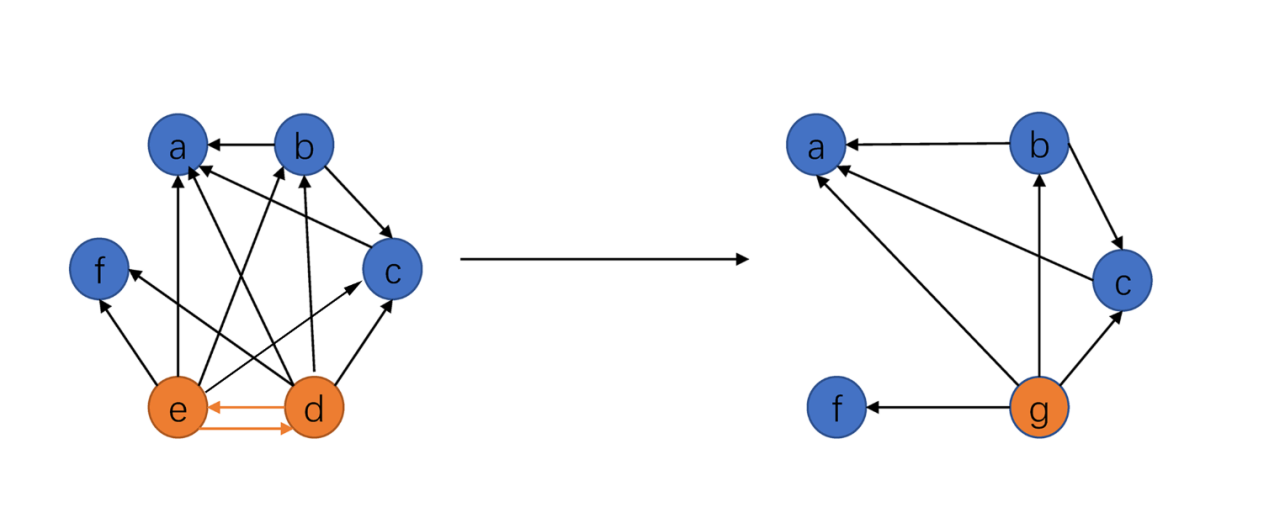


*G_ij_*  *G_ij_^*^*

**Figure S5** The construction of graphs *G_ij_* and $G_{ij}^{*}$. Following the rules mentioned in main text, the graph *G_ij_* is constructed under all columns using rows *i* and *j,* and the acyclic graph$G_{ij}^{*}$ is obtained by contracting the clique which consists of two nodes *d* and *e* into a new node *g*.


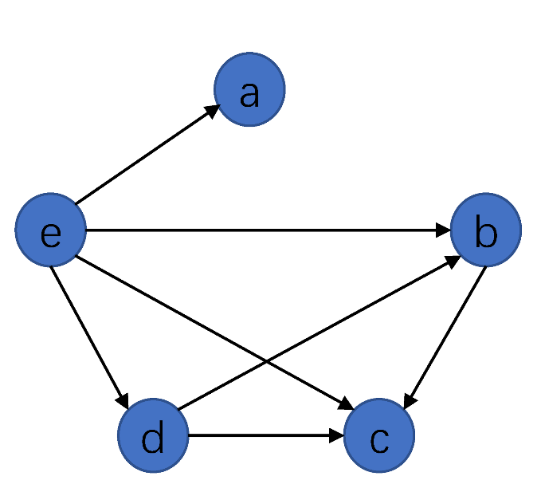


*G_ik_*

**Figure S6** The construction of graph *G_ijk_* using rows *i* and *k*. Following the rules mentioned in main text, the graph *G_ijk_* is constructed on the nodes/columns (*a*, *b*, *c*, *d*, *e*) based on the rows *i* and *k* in the same way as used in constructing *G_ij_*.

1. **ARBic outperformed the existing tools for finding biclusters with different patterns**


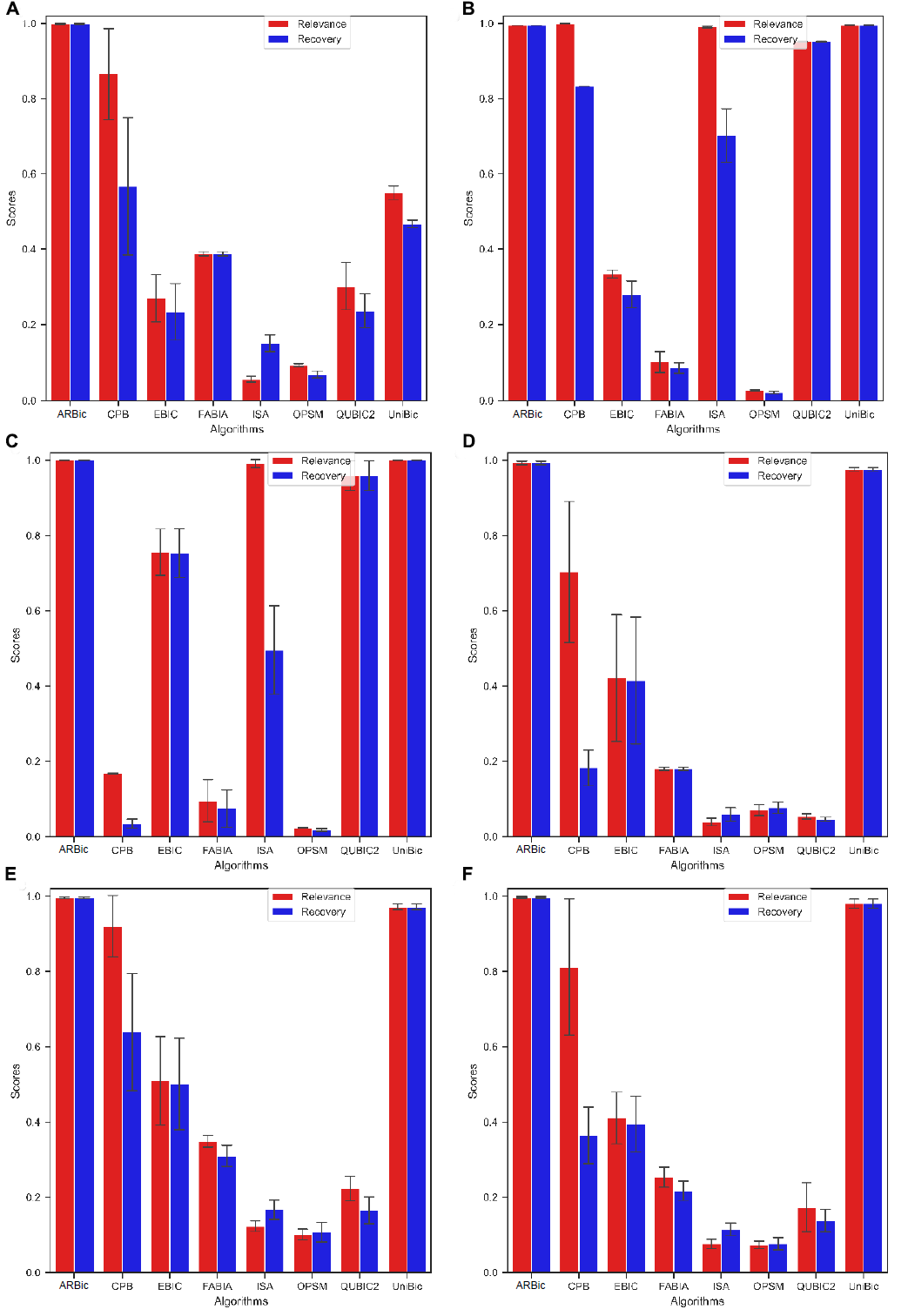


Figure S7 Comparisons of the eight tools on the six datasets containing various bicluster patterns. a) Results on the dataset implanted with trend-preserving biclusters. b) Results on the dataset implanted with column constant biclusters. c) Results on the dataset implanted with row constant biclusters. d) Results on the dataset implanted with scale biclusters. e) Results on the dataset implanted with shift biclusters. f) Results on the dataset implanted with shift-scale biclusters.

1. **Details of real datasets used in the maintext**

Table S1. The summary of datasets used in the study

| **Datasets** | **#genes** | **#conditions** | **Download links** |
| --- | --- | --- | --- |
| **E. coli Colombos** | **2093** | **2470** | **https://doi.org/10.5281/zenodo.1157938** |
| **E. coli DREAM5** | **2442** | **805** | **https://doi.org/10.5281/zenodo.1157938** |
| **Yeast GPL2529** | **3178** | **3025** | **https://doi.org/10.5281/zenodo.1157938** |
| **Yeast DREAM5** | **3292** | **536** | **https://doi.org/10.5281/zenodo.1157938** |
| **Human SEEK GPL5175** | **4436** | **2308** | **https://doi.org/10.5281/zenodo.1157938** |
| **Pathwasys** |  |  | **https://doi.org/10.5281/zenodo.1157938** |

1. **Details of parameters used in the main text**

Table S2. The summary of parameters used in the study

| **Algorithms** | **Simulation data** | **Real data** |
| --- | --- | --- |
| **ARBic** | **o = NO.** | **r_range=[1, 8 ,15]**  **c_range=[0.7,0.8,0.9]**  **f_range=[0.5,0.7,0.9]** |
| **CPB** | **n = NO.** | **nclus=[40,41,42,43,44,45,46,48,49,**  **50,51,52,53,54,55,56,57,58,59]** |
| **EBIC** | **b = NO., n=5000** | **n=[1000,2000,3000,5000]**  **x=[0.5,0.6,0.7,0.85,0.9]** |
| **FABIA** | **p = NO.** | **alpha=[0.1,0.2,0.3,0.4,0.5]**  **spl=[0.5,1.0]**  **spz=[0.5,1.0]** |
| **ISA** | **no_seeds = NO.** | **No_seeds=[10,20,30,40,50,60,70,80,90**  **100,110,120,130,140,150,160,170,180,190]** |
| **OPSM** | **lValue = NO.** | **lValue=[40,41,42,43,44,45,46,47,48,49**  **50,51,52,53,54,55,56,57,58,59]** |
| **QUBIC2** | **q = 0.06 - 0.5** | **r_range=[1, 8 ,15]**  **c_range=[0.7,0.8,0.9]**  **f_range=[0.5,0.7,0.9]** |
| **UniBic** | **o = NO.** | **r_range=[1, 8 ,15]**  **c_range=[0.7,0.8,0.9]**  **f_range=[0.5,0.7,0.9]** |

1. **Data preprocessing step**

In the regulation of gene expression, the expression level of genes in an up-regulated state will be higher than that of genes that are normally expressed. The expression level of genes in a down-regulated state will be lower than that of normally expressed genes. Generally speaking, when analyzing gene expression data, differentially expressed genes have more biological significance. In order to bicluster the differential expression values, we need to preprocess the input data.

For the expression value of a gene under all conditions, we take the median of gene expression under all conditions as the normal expression value of the gene. Then the expression value of the gene up-regulated (down-regulated) is the expression value far away from the normal expression level (median). Let $q$ be the ratio of what we think of as up-regulation and down-regulation expression values. The selection process of upper and lower data is as follows:

1. For any gene, the expression levels of the gene are in non-strict ascending order according to the expression value (the expression level) as $a_{i,1}$*…*$a_{i,s}\ldots a_{i,l}\ldots a_{i,n-s}$*,…*$a_{i,n}$,where $l=\frac{m}{2}, s=nq$.

2. Let $d=min(a_{i,l}-a_{i,s}$*,*$a_{i,n-s}-a_{i,l})$, the gene expression of our behavior is divided into three expression intervals $\left[ a_{i,1},a_{i,l}-d \right]$,$\left( a_{i,l},-d{,a}_{i,l}+d \right)$,$\left[ a_{i,l}+d,a_{i,m} \right]$, respectively represent the expression interval of the gene, the normal expression interval and the expression interval.

3. Set the expression values in the interval $\left( a_{i,l},-d{,a}_{i,l}+d \right)$ as 0, which means we ignore the normal expression of the gene.

The existence of experimental errors causes some errors in gene expression values. In order to reduce the influence of errors on bi-clustering, we adopt a grouping strategy to preprocess the expression data.

We equally partition all the up-regulated expression values of a gene in decreasingly ordered into r intervals, and we set the expression values belonging to the ith interval to be the integer i. We equally partition all the down-regulated expression values of a gene in increasingly ordered into r intervals, and we set the expression values belonging to the ith interval to be the integer -i.

1. **Average running time of algorithms**

Table S3 Average running time of algorithms

| Time (s)  Algorithms  Datasets | ARBic | CPB | FABIA | ISA | OPSM | EBIC | QUBIC2 | UniBic |
| --- | --- | --- | --- | --- | --- | --- | --- | --- |
| E. coli Colombos | 1210 | 66 | 367 | 183 | 5838 | 150 | 19 | 5 |
| E. coli DREAM5 | 1161 | 15 | 148 | 246 | 2499 | 155 | 9 | 241 |
| Yeast GPL2529 | 9157 | 116 | 673 | 1159 | 12753 | 175 | 67 | 197 |
| Yeast DREAM5 | 633 | 17 | 131 | 335 | 1866 | 177 | 8 | 271 |
| Human SEEK GPL5175 | 13873 | 123 | 676 | 180 | 12260 | 177 | 39 | 1191 |

1. **experiments on seqgendiff simulated data**

We find that seqgendiff is a well-designed tool to mimic patterns in real world data. So we compare performance of all algorithms on the seqgendiff simulated data. We choose the GTEx muscle data with genes with a mean read-depth of greater than 10 reads as base real data. We use seqgendiff to thin and then generate simulated trend-preserving data from the real data. ARBic still is the best algorithm.


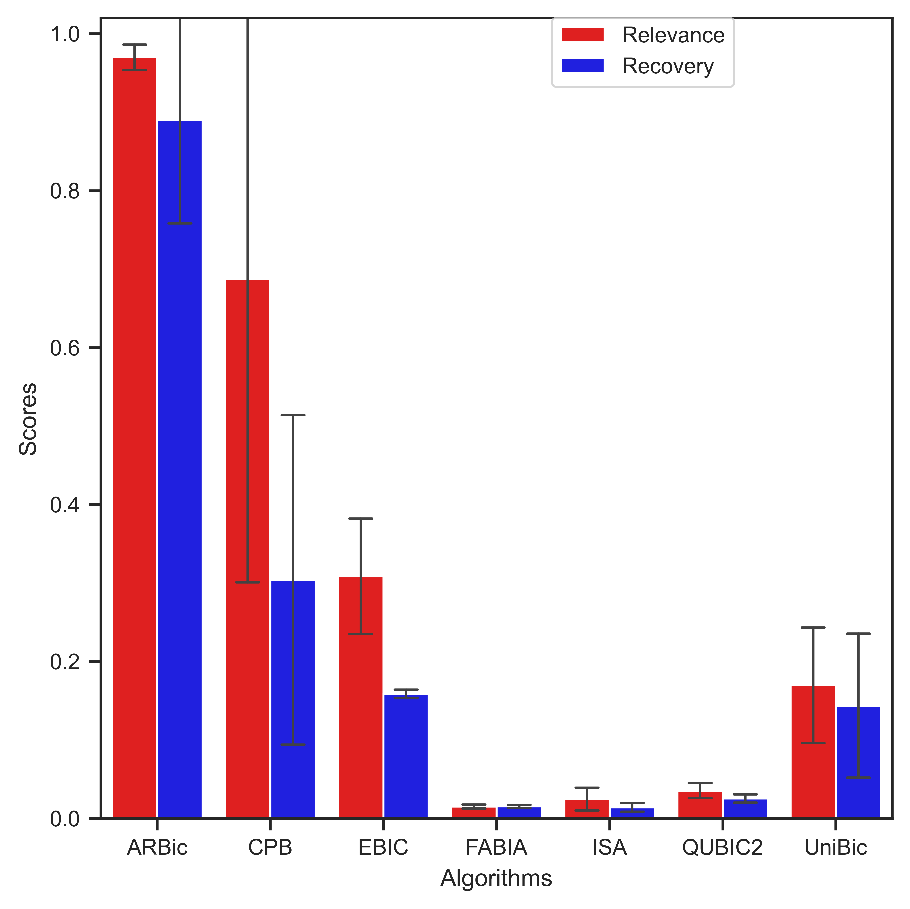


Figure S8 relevance and recovery scores on seqgendiff simulated data.

1. **Heatmap of BC00 and BC01**

We show bicluster BC00 and BC01 found in E. coli Dream5 as an example. From the heatmap, we can see that each gene tends to change its color from blue to yellow in a same bicluster, implying that their expressions are trend-preserving. We can see the BC00 is enriched by KEGG PATHWAY: eco02040 which is Flagellar assembly - Escherichia coli K-12 MG1655 (belonging to the class: Cellular Processes; Cell motility) and BC01 was enriched by KEGG PATHWAY: eco03010 which is the Ribosome - Escherichia coli K-12 MG1655 PATHWAY(belonging to the class: Genetic Information Processing; Translation).


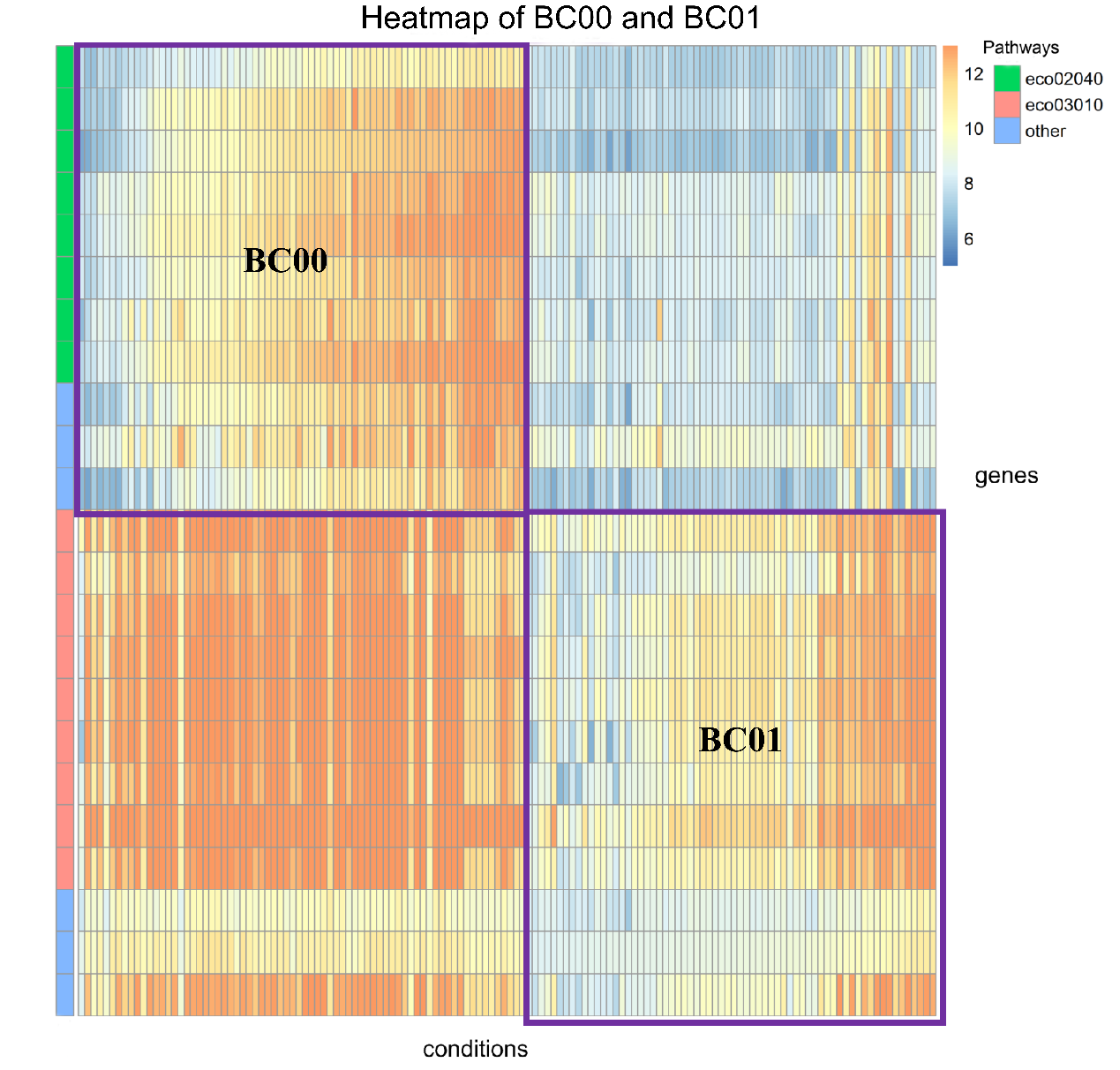


Figure S9 heatmap of BC00 and BC01 found by ARBic.

**References:**

1. Ben-Dor, A., et al., *Discovering local structure in gene expression data: the order-preserving submatrix problem.* Journal of computational biology, 2003. **10**(3-4): p. 373-384.
